# Active layers of high-performance lead zirconate titanate at temperatures compatible with silicon nano- and microelecronic devices

**DOI:** 10.1038/srep20143

**Published:** 2016-02-03

**Authors:** Iñigo Bretos, Ricardo Jiménez, Monika Tomczyk, Enrique Rodríguez-Castellón, Paula M. Vilarinho, M. Lourdes Calzada

**Affiliations:** 1 Instituto de Ciencia de Materiales de Madrid (ICMM), Consejo Superior de Investigaciones Científicas (CSIC), Sor Juana Inés de la Cruz 3, Cantoblanco, Madrid 28049, Spain; 2 Department of Materials and Ceramic Engineering & CICECO, University of Aveiro, Aveiro 3810-193, Portugal; 3 Departamento de Química Inorgánica, Cristalografía y Mineralogía, Facultad de Ciencias, Universidad de Málaga, Málaga 29071, Spain

## Abstract

Applications of ferroelectric materials in modern microelectronics will be greatly encouraged if the thermal incompatibility between inorganic ferroelectrics and semiconductor devices is overcome. Here, solution-processable layers of the most commercial ferroelectric compound ─ morphotrophic phase boundary lead zirconate titanate, namely Pb(Zr_0.52_Ti_0.48_)O_3_ (PZT) ─ are grown on silicon substrates at temperatures well below the standard CMOS process of semiconductor technology. The method, potentially transferable to a broader range of Zr:Ti ratios, is based on the addition of crystalline nanoseeds to photosensitive solutions of PZT resulting in perovskite crystallization from only 350 °C after the enhanced decomposition of metal precursors in the films by UV irradiation. A remanent polarization of 10.0 μC cm^−2^ is obtained for these films that is in the order of the switching charge densities demanded for FeRAM devices. Also, a dielectric constant of ~90 is measured at zero voltage which exceeds that of current single-oxide candidates for capacitance applications. The multifunctionality of the films is additionally demonstrated by their pyroelectric and piezoelectric performance. The potential integration of PZT layers at such low fabrication temperatures may redefine the concept design of classical microelectronic devices, besides allowing inorganic ferroelectrics to enter the scene of the emerging large-area, flexible electronics.

Ferroelectric oxides are materials of high interest in electronics owing to their inherent multifunctionality in advanced devices making use of their ferro-, pyro-, piezo- and optoelectronic properties^1^. The major obstacle for the integration of these active layers with CMOS (complementary metal-oxide semiconductor) circuits lies on the high processing temperatures required for the crystallization of the ferroelectric oxide, over 600 °C. The thermal budget currently recommended for manufacturing sub-100 nm semiconductor devices sets a maximum limit of temperature of 450 °C^2^. Higher thermal loads may produce excessive dopant diffusion from the source/drain, leading to a short-channel effect that degrades transistor performance and manufacturability. In addition, ferroelectric oxide thin films (mainly lead-based and bismuth-layered perovskite compositions) are considered a potential extra-source of pollution due to the volatilization of high-vapor pressure elements such as Pb and Bi at the temperatures required for the film fabrication^3^. Metal contamination is a well-known problem in the standard CMOS process that results in circuitry degradation and cross-contamination of back end of the line equipment. Besides the technological issue, the release of hazardous lead towards the atmosphere is also a global subject of environmental concern^4^, being of high interest the development of new processing technologies that avoid the emission of toxic volatiles. Therefore, the reduction of the annealing temperature of ferroelectric oxide films is still a key challenge for their full integration into Si-based semiconductor chips.

Although significant efforts have been devoted to the low-temperature processing of ferroelectric oxide thin films using different techniques, the limited success obtained at the moment (UV-assisted annealing^5^,^6^,^7^,^8^,^9^,^10^, laser annealing process^11^,^12^, microwave irradiation^13^,^14^, seeding approach^15^,^16^, process engineering^17^−^19^ or most recent heterogeneous photocatalysis^20^) reveals the big complexity of this task. Special attention has been paid to chemical solution deposition (CSD) methods since – among other benefits such as comparatively low cost, compositional control, and high-throughput fabrication – they offer the particular advantage of tailoring the solution chemistry to attain a low-temperature fabrication of these materials^21^,^22^,^23^,^24^. Although coating of 3D structures can also be obtained from liquid precursors by mist deposition^25^, most electronic devices with commercial success fabricated by CSD^26^, display a planar ferroelectric-capacitor configuration that finds important applications in multiple fields as sensors (e.g., infrared detectors), transducers (e.g., fingerprint scanners), actuators (e.g., ultrasonic micromotors), or non-volatile memories (e.g., smart cards). The latest ITRS report^2^ predicts the use of 1T1C (one-transistor, one-capacitor) stack cells in FeRAM (ferroelectric random access memory) technology up to 2028, a device structure where CSD may play an important role for low memory density products.

The road to the thermal compatibility between inorganic ferroelectrics and the standard CMOS process of semiconductors is however not straightforward. The decrease of the annealing temperature of the oxide layers results in incipient crystallization of the required crystalline phases (besides stabilization of detrimental secondary phases) and limited densification of the film, risking poor electrical and mechanical performance. These handicaps are today circumvented only by a few approaches, most of them entailing technical drawbacks for a potential integration into CMOS devices. For instance, laser lift-off techniques without any growth temperature limitation are successful in transferring ferroelectric thin films to any type of substrate, but these result in costly process besides adding an enormous complexity to it^27^. The crystallization temperature of ferroelectric thin films can be reduced by their direct growth on a seed layer of a dissimilar material displaying the same crystalline structure (i.e., by lowering the activation energy for perovskite nucleation)^28^. Buffer layers of dielectric SrTiO_3_, PbTiO_3_ and SrRuO_3_, or conductive LaNiO_3_ oxides have been employed to obtain Pb(Zr_x_Ti_1-x_)O_3_ ferroelectric thin films at annealing temperatures close to 450 °C^11^,^29^,^30^,^31^. However, it is worth noting that high temperatures (500–700 °C) are previously required to crystallize the seed layer on the silicon substrate, which ruins the efforts to not degrade the semiconductor transistor apart from increasing the complexity of the integration process. Particularly for lead- and bismuth-based perovskite films, a strategy used to decrease their preparation temperature consists in the incorporation of either PbO or Bi_2_O_3_ excesses, even up to 50 mol%, to the respective precursor systems^18^,^32^. These compounds enhance (as a flux for crystal growth) the solid-state diffusion of elements during crystallization, leading to the formation of the ferroelectric crystal phase at lower temperatures with respect to stoichiometric samples^33^,^34^. On the other hand, the evaporation of elemental Pb and Bi is known to occur earlier (i.e., at lower temperatures) from the respective excess (amorphous) oxides rather than from the nominal perovskite phase^35^. Volatilization of such species at these processing conditions results in the detrimental contamination of conventional circuits at the back equipment.

Apart from the former crystallization aids, the fabrication temperature of ferroelectric oxide films can also be reduced intrinsically to the chemical composition of the material candidate. Thus, the crystallization temperature of the PbO-ZrO_2_-TiO_2_ solid solution decreases as the Zr/Ti ratio is lowered^28^. In other words, the Ti-rich compositions are the ones that can be crystallized at a lower temperature, with minimum values reported for ferroelectric Pb(Zr_0.30_Ti_0.70_)O_3_ films around 400 °C^19^. The former composition of tetragonal symmetry is at present the best material choice for the development of ferroelectric memories by companies. We must strengthen that Pb(Zr_x_Ti_1-x_)O_3_ with a composition near the morphotropic phase boundary (MPB), namely Pb(Zr_0.52_Ti_0.48_)O_3_, displays a crystallization temperature between 500–700 °C that has historically hampered its preparation at temperatures suitable for the current CMOS technology. This material is certainly the ferroelectric with the greatest commercial applicability, and the basis of the current high-sensitivity piezoelectric ceramics^36^. Therefore, its fabrication at temperatures compatible with semiconductor devices would represent a major breakthrough in the field of nano- and microelectronics, covering a potential industry need for the coming years.

In this work, we demonstrate the high performance of Pb(Zr_0.52_Ti_0.48_)O_3_ (hereinafter, PZT) thin films grown on silicon substrates at 400 °C, an annealing temperature consistent with the standard process followed in the semiconductor industry for embedding into logic circuits. A low-temperature solution method is used whereby neither modification of substrate heterostructure (by incorporation of seed layers) nor the addition of PbO excess is needed, thus avoiding technological drawbacks to the integration routine. The evaluation of the ferro-, pyro-, and piezoelectricity of these low-temperature PZT films on silicon reveals the multiple functionality that this active layer offers in a wide range of advanced electronic devices such as FeRAMs, ferroelectric field effect transistors (FeFETs), uncooled infrared sensors, or micro/nano-electromechanical systems (transducers, actuators).

## Results and Discussion

### Solution systems and crystallization mechanisms

**Figure 1:**
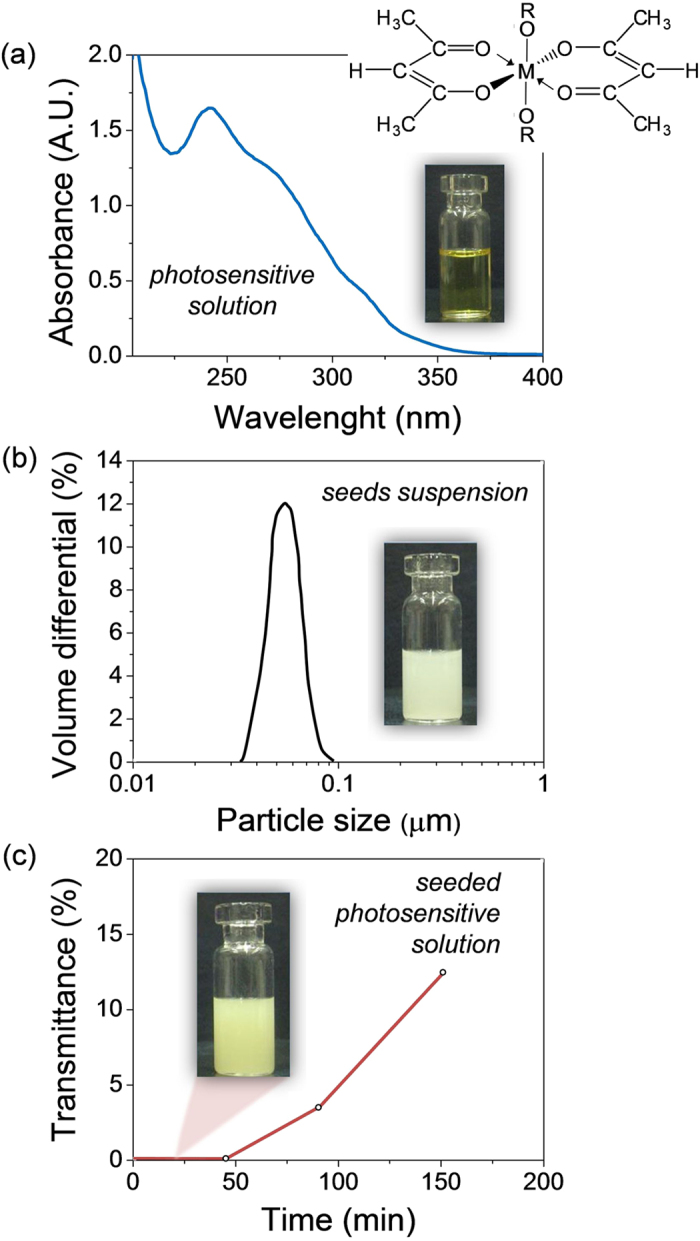
Physicochemical properties of the PZT precursor solutions. (**a**) UV absorption of the *Photosensitive* solution, where both Zr and Ti alkoxides have been stabilized with acetylacetone (see general molecular structure, where M = metal atom and R = organic substituent). (**b**) Particle size distribution of a seeds dispersion constituted by PZT nanopowders in ethanol. (**c**) Stability with time of the *Seeded Photosensitive* solution measured by light transmittance.

The low-temperature processing of the PZT thin films of this work was carried out by photochemical solution deposition (PCSD)^9^,^10^,^37^. This method consists in the synthesis of a photosensitive precursor solution (hereinafter, *Photosensitive* solution) that, after deposition on a substrate, leads to a gel film susceptible of photoactivation by UV irradiation. At this step, the electronic excitation of particular chemical bonds is produced in the system, which enhances the decomposition of the metal precursors promoting the crystallization of the oxide phase at relatively low temperatures^38^. Additionally, the irradiation is carried out in oxygen atmosphere leading to the formation of ozone (O_3_) and singlet oxygen species O(^1^D). The former contributes to the further oxidation of organic species, while the latter reacts with suboxides present in the film thus improving the stoichiometry and decreasing the density of defects and oxygen vacancies^37^,^39^. To provide additional nucleation sites in the film that facilitate the early stages of crystallization^16^,^40^, perovskite nanoseeds of PZT were introduced in this solution obtaining a seeded diphasic photosensitive precursor (hereinafter, *Seeded Photosensitive* solution). The main physicochemical properties of both solution systems are shown in Fig. 1. The *Photosensitive* solution shows an absorption maximum at ~240 nm (Fig. 1a) that corresponds to the π → π* electronic transition developed by the acetylacetonate (acac) molecules^41^, present in both titanium and zirconium alkoxide precursors (see Experimental Section). Perovskite nanoseeds were introduced in this solution to prepare a seeded diphasic precursor (see Experimental Section). An average size of ~50 nm is measured for the constituent particles of a suspension of PZT nanopowders in ethanol (Fig. 1b). The light transmittance measured in the resulting *Seeded Photosensitive* solution did not increase during the first 45 min (Fig. 1c), which indicates that the mixture obtained is stable for this time.

**Figure 2:**
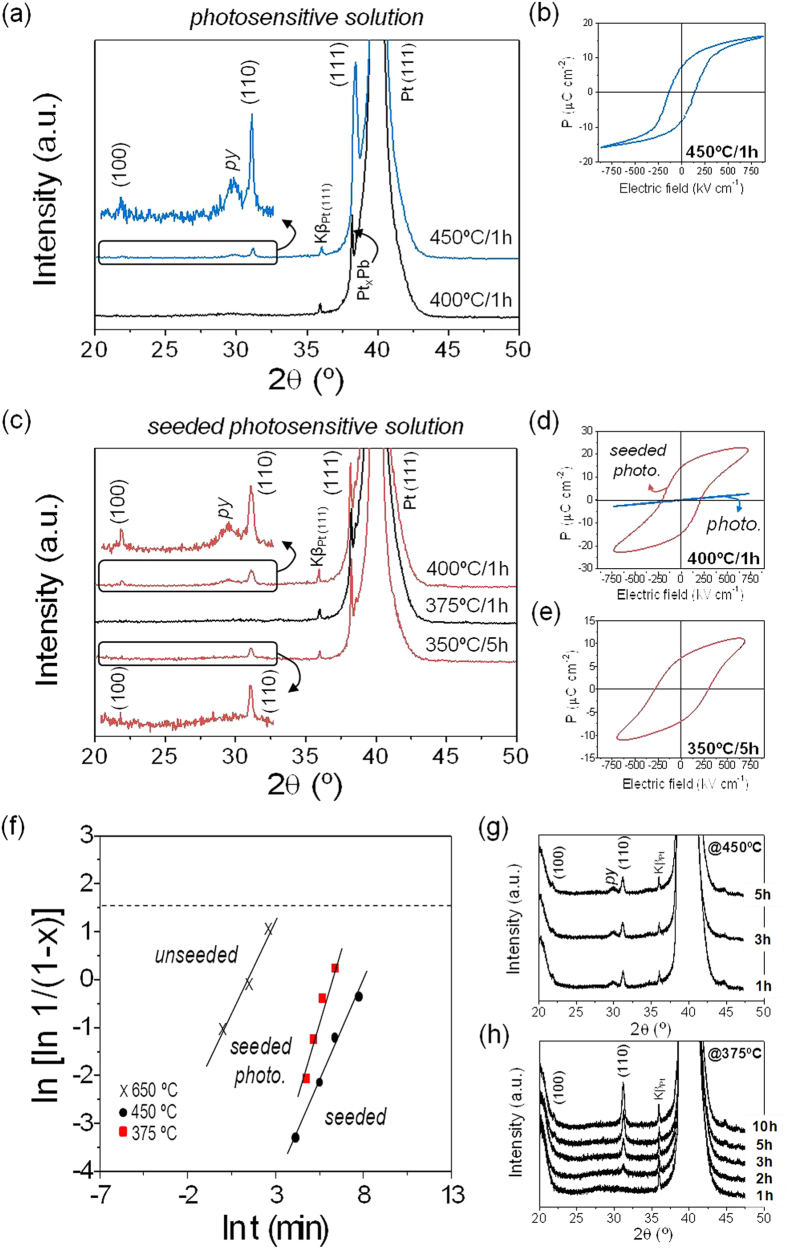
Perovskite formation and crystallization kinetics of the PZT thin films. The minimum annealing temperature required to obtain perovskite films with ferroelectric response from the *Photosensitive* (**a,b**), and *Seeded Photosensitive* (**c–e**) solution systems is shown (py = pyrochlore). Magnifications in the XRD patterns are provided for a better observation. (**f**) Avrami plots showing the evolution of the perovskite phase with annealing time are depicted for films crystallized at different temperatures from the *Seeded Photosensitive* solution, and from conventional unseeded and seeded solutions. XRD patterns of the films prepared from the *Photosensitive* solution at 450 °C (**g**) and *Seeded Photosensitive* solution at 375 °C (**h**) for different annealing times.

A comprehensive analysis of the crystallization process developed in 70 nm thick PZT films prepared from the former solution systems is shown in Fig. 2. The minimum annealing temperature required for each system to obtain a crystalline film with a demonstrated functionality (through the corresponding ferroelectric hysteresis loop) is highlighted. Thus, reflections corresponding to the perovskite structure with a minor pyrochlore phase are detected at 450 °C in the films derived from the *Photosensitive* solution (Fig. 2a). The intense peak at ~38.4° (2θ) indicates the PZT growth with a < 111 > preferred orientation. However, a contribution of intermetallic Pt_x_Pb phases to this peak should not be excluded^42^. These are transient compounds formed at low temperatures between the PZT film and the metal electrode of the substrate, promoting the appearance of such texture in the films^43^,^44^. The reflection of this intermetallic phase appears at ~38.1° (2θ) in the film annealed at lower temperature (400 °C), where no reflection corresponding to the perovskite phase is observed (i.e., amorphous PZT film). Therefore, a ferroelectric response in the *Photosensitive* solution derived films (Fig. 2b) is only reached from 450 °C, an annealing temperature in the maximum limit of current CMOS devices. Note that ferroelectric PZT films are achieved here in the absence of lead excess, which is a remarkable difference from the few publications reporting to date MPB-PZT thin films annealed at 450 °C showing ferroelectricity^13^,^14^. The possible volatilization of this element during annealing is thus minimized here, avoiding the risk for metal contamination in the manufacturing process of electronic components on semiconductors. On the other hand, a further decrease of the crystallization temperature is observed in the PZT films prepared from the *Seeded Photosensitive* solution (Fig. 2c). Perovskite reflections are now detected at an annealing temperature of 400 °C, as deduced from the respective X-ray pattern of the film. The presence of crystalline nanoseeds in this system decreases the energy barrier for the perovskite nucleation, leading to a significant reduction in the preparation temperature of PZT films with ferroelectric properties^45^. Note that a linear (non ferroelectric) dielectric response is obtained when PZT films from the *Photosensitive* solution are prepared at the same temperature (Fig. 2d). This reduced thermal budget may bring the FeRAM technology closer to the 65-nm node (and beyond) of semiconductor manufacturing, resulting in a product with less power consumption that provides both economical and environmental benefits to the integration process^46^. A decrease down to 350 °C can even be achieved by the application of longer dwell times (5 h). This result can be explained from a kinetic perspective using the Avrami phase transformation expression^47^, which correlates the volume fraction of the perovskite phase (x) developed with the annealing time (t). Figure 2f shows Avrami plots for several PZT thin films annealed at different conditions of temperature and time. Samples from the *Seeded Photosensitive* solution are compared with those derived from unseeded and seeded solutions^45^. The relatively high slope of the fitting line obtained for the film derived from the former solution indicates that the perovskite phase grows in this system even at a faster rate with respect to films derived from conventional solutions annealed at high temperatures (650 °C). The enhanced decomposition of metal precursors by UV-light accounts for the rapid crystallization of the perovskite phase in the film provided a number of crystalline nuclei are previously formed. It is worth pointing out that despite nucleation of the perovskite phase being achieved in films derived from *Photosensitive* solutions at 450 °C, coexistence with the secondary pyrochlore phase is observed even up to long annealing times (see Fig. 2g). Irradiation by UV-light enables the formation of an amorphous but highly reactive precursor that crystallizes into the oxide phase at much earlier stages (i.e., lower temperatures) than usual. However, this photoactivation is not as high as to surpass the energetic barrier for the nucleation of those secondary phases that are stable in this oxide system at these low temperatures. As a consequence, perovskite and pyrochlore coexist in these films. The addition of perovskite nanoseeds to this solution (*Seeded Photosensitive* system) induces the crystallization of the perovskite phase without stabilization of the pyrochlore one (see Fig. 2h). In addition, to get an almost fully-crystallized perovskite material (see dashed line corresponding to x = 0.99) using a low-temperature process, the films derived from the *Seeded Photosensitive* solution would require a much lower thermal budget (375 °C for 30 h) with respect to those obtained from seeded solutions (450 °C for 300 h). This result supports the integration of ferroelectric oxide layers not only with CMOS-compatible semiconductor devices2 but also into next-generation flexible electronics^48^ based on polymeric substrates with thermal stability below the thermal border of 400 °C.

**Figure 3:**
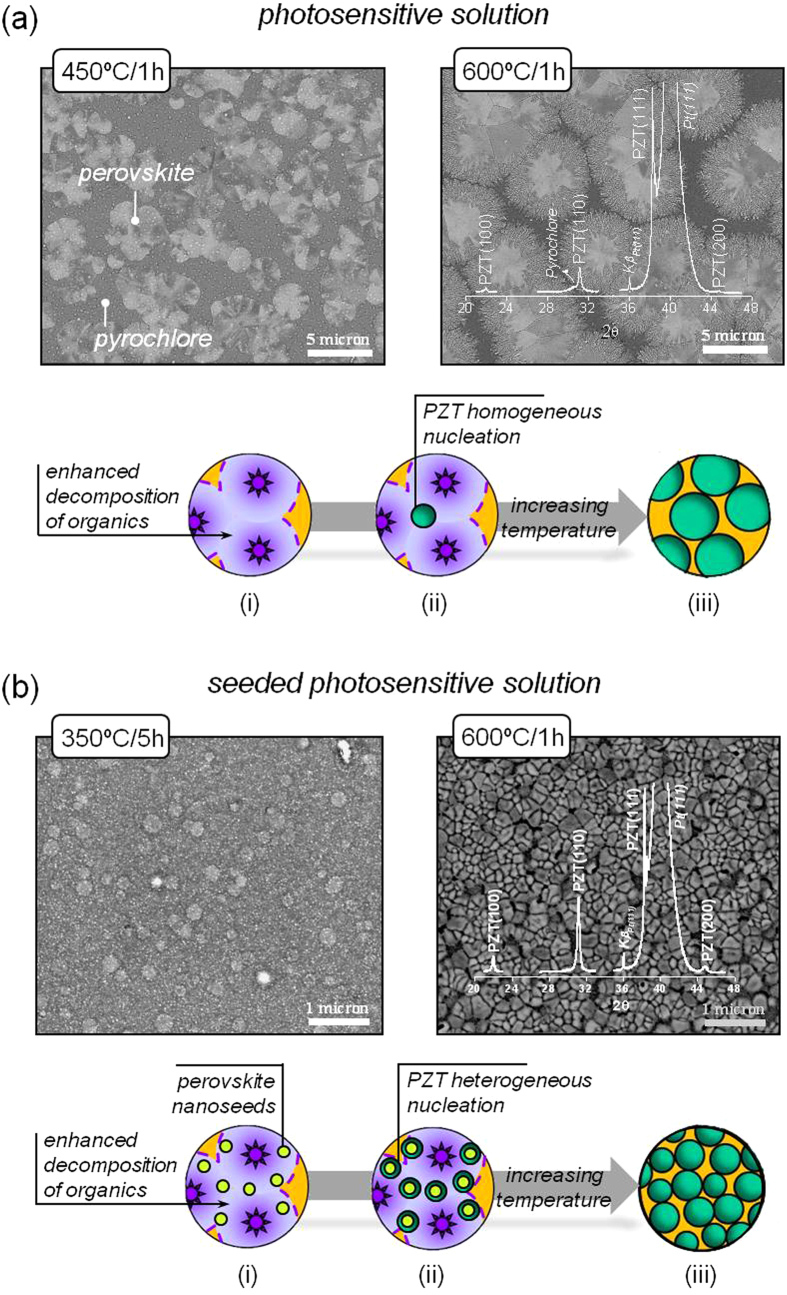
Surface morphology by SEM of the PZT thin films obtained at different annealing temperatures from the *Photosensitive* (**a**) and *Seeded Photosensitive* (**b**) solution systems. X-ray diffractograms of the films annealed at 600 °C are included. A scheme showing the respective crystallization mechanisms proposed for each system at different stages (*i*: photoactivated gel film; *ii*: amorphous oxide film; *iii*: crystalline PZT film) is depicted.

Figure 3 displays the surface morphology revealed by scanning electron microscopy (SEM) of the PZT films prepared from the two different solution systems of this work. Images corresponding to films derived from the *Photosensitive* (Fig. 3a) and *Seeded Photosensitive* (Fig. 3b) solutions annealed at different temperatures are shown. In the first case, morphologically distinct features are observed in the 450 °C-annealed film that can be associated with the coexistence of perovskite and pyrochlore phases detected in the respective XRD analysis of Fig. 2a. Perovskite grains displaying rosette-type morphology appear immersed within a fine-grain matrix of a secondary phase (pyrochlore). When these films are subjected to a higher temperature (600 °C), the surface microstructure evolves towards a majority perovskite phase in which intergranular pockets of pyrochlore still remain. The topology obtained suggests a radial growth with temperature of the perovskite rosettes until they impinge on one another^49^. The XRD pattern of this film denotes a clear <111> preferred orientation, with a relative intensity for the *I*(111)/*I*(101) reflections of 100/37. It is generally accepted that the resulting texture in this system arises from the heterogeneous nucleation of perovskite crystals at the Pt electrode of the substrate^50^. On the other hand, the perovskite film prepared from the *Seeded Photosensitive* solution at 350 °C shows a uniform surface morphology consisting of nanosized grains with a mean diameter of ∼30 nm (see Fig. 3b). This microstructure would be suitable for the reliable fabrication of discrete capacitors with a low aspect ratio, such as FeRAMs. These grains grow moderately with the annealing temperature (30–50 nm), and the surface morphology obtained in the 600 °C-annealed PZT film is highly representative of a polycrystalline ceramic material. A single perovskite phase with a relatively lower <111> preferred orientation is inferred from the corresponding pattern. Note that the relative intensity for the *I*(111)/*I*(101) reflections is now of 100/60, denoting the larger contribution of randomly oriented crystals to the perovskite structure of this film. This effect is related to the presence of the crystalline nanoseeds in the films derived from the *Seeded Photosensitive* solution. Heterogeneous nucleation not only at the substrate interface, but also at the surface of the seeds occurs now in these films. While the former mechanism is usually responsible for the <111> texture, the nucleation on the perovskite nanoseeds yields the additional <101> component also observed in the films. These results, together with the microstructural evolution with temperature of the films displayed in this figure, allow us to draw their respective crystallization mechanisms relative to the different solution systems. A schematic of them appears respectively depicted at the bottom of the SEM images. The fine-grained morphology obtained for the low-temperature thin films derived from the *Seeded Photosensitive* solution corresponds well to a high density of nucleation sites within the amorphous layer, in this case, ascribed to the presence of perovskite nanoseeds. This effect results in the formation at higher temperatures of polycrystalline microstructures constituted by a large number of equiaxed grains. The lack of seeds in the films derived from the *Photosensitive* solution would limit the crystallization event only to a few nuclei, here described as isolated rosettes, in which grain growth with temperature leads to crystalline films formed by significantly larger grains.

**Figure 4:**
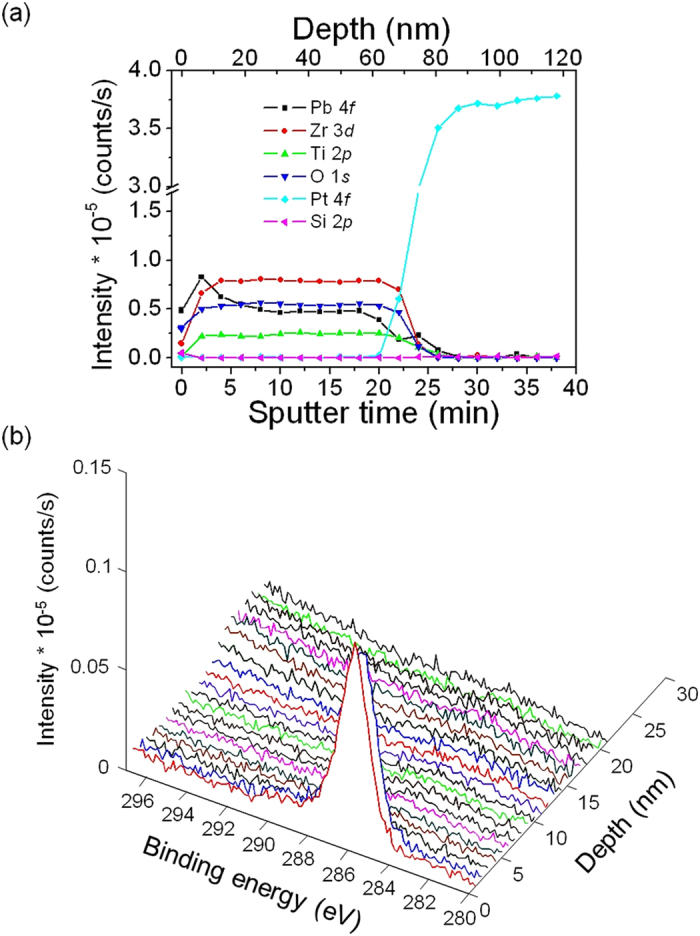
Compositional depth profile by XPS of the PZT thin film prepared from the *Seeded Photosensitive* solution at 400 °C. (**a**) Photoelectron peak intensities of constituent elements of the sample during depth profiling. (**b**) Evolution of the C 1*s* core level signal as a function of depth and binding energy.

The different mechanisms proposed for the perovskite formation in each system accounts for the crystallization temperature of the corresponding PZT films, as well. As a rule of thumb, the higher the number of nuclei of the perovskite phase, the lower the crystallization temperature of the ferroelectric film is. The electronic excitation of the photoactive species present in the gel films by UV irradiation provides a high-energy region where the decomposition of organic compounds is enhanced (see schemes of Fig. 3), thus promoting crystallization at low temperatures in both systems. However, the large density of nucleation sites in the films derived from the *Seeded Photosensitive* solution induces the development of a greater number of perovskite nuclei in this system and inhibits the stabilization of the pyrochlore phase with respect to the *Photosensitive* solution, where both perovskite and pyrochlore coexist. As a consequence, lower annealing temperatures are required to obtain ferroelectric PZT films from the first system, in agreement with the XRD results shown in Fig. 2. Despite the low processing temperature used, complementary analysis by X-ray photoelectron spectroscopy (XPS) revealed that the presence of retained carbon in these films is null. Figure 4 shows the XPS depth profile of the PZT thin film prepared from the *Seeded Photosensitive* solution at 400 °C. After several minutes of Ar^+^ bombardment, a steady-state situation is reached in the sample whereby a homogeneous profile is deduced for the constituent elements of the film (Fig. 4a). Additionally, the non corrected C 1*s* core level spectrum (Fig. 4b) before sputtering with Ar^+^ denotes a signal at ~286 eV corresponding to adventitious carbon that vanishes after 1 min of sputtering time. This phenomenon is commonly attributed to the surface accumulation of carbonaceous contaminants from the ambient (e.g., graphitic carbon, hydrocarbons, carbonates) on the sample prior to the experimental analysis^42^. Once the sputtering cleaning begins, these species are completely removed from the sample, neither detecting carbon residuals in the bulk of the film.

### Functionality of the low-temperature thin films

The promising integration of ferroelectric oxide layers into the ubiquitous technology of silicon semiconductors cannot be solely reduced to a thermal compatibility issue. Several works have reported the low-temperature crystallization of perovskite films comprised of a ferroelectric oxide composition indeed. However, demonstration of the functionality of these materials through the measurement of a ferroelectric response is often missed. The main reason usually lies in the stabilization of detrimental secondary phases (pyrochlore, fluorite) by short-range diffusion at such low temperatures, restricting the complete crystallization of the perovskite phase^51^,^52^. In the most favourable case, an incipient perovskite phase can appear inmersed into a matrix of these non-ferroelectric compounds that cancel the ferroelectric response of the material. Therefore, the potential use of ferroelectric thin films as memories, sensors or actuators in advanced electronic devices primarily involves an optimum working performance, besides fulfilling the processing steps (temperature control, uniformity, limiting damage and contamination) of CMOS technology.

**Figure 5:**
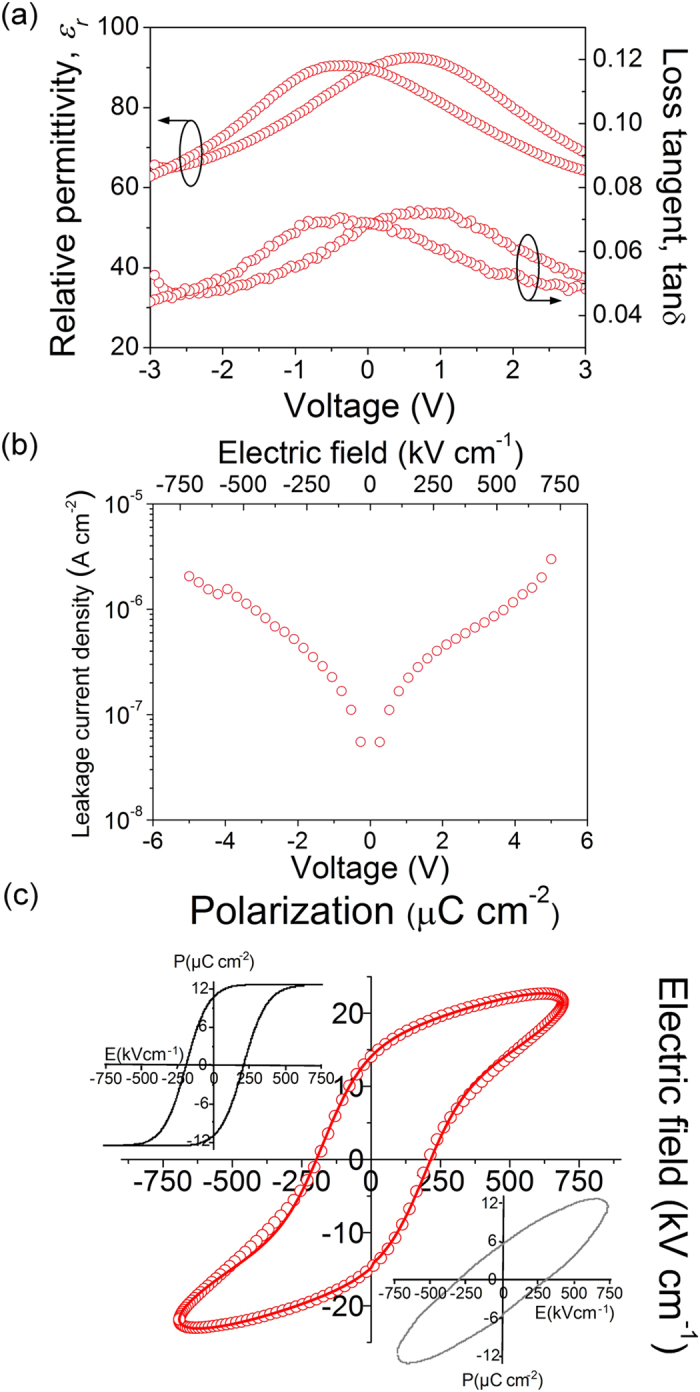
Dielectric and ferroelectric properties at room temperature of the PZT thin film prepared from the *Seeded Photosensitive* solution at 400 °C. (**a**) Variation of the relative permittivity and loss tangent with bias voltage. (**b**) Leakage current density as a function of applied voltage and electric field. (**c**) Experimental hysteresis curve measured in the film including the pure ferroelectric loop (up left) and non-switching contribution (down right) of the sample.

The dielectric and ferroelectric response of the PZT thin films prepared from the *Seeded Photosensitive* solution at 400 °C is evaluated in Fig. 5. The variation of the relative permittivity (ε_r_) and loss tangent (tanδ) with bias voltage shown in Fig. 5a evidences the hysteretic and non-linear nature of these films. The two distinctive maxima in the curve of the respective loops are indicative of ferroelectric domain switching. A value for the relative permittivity of ~90 is measured at zero voltage, with dielectric losses below 10%. Lower permittivity values are obtained with respect to PZT films annealed at conventional temperatures (600–700 °C) suggesting that the crystal quality and microstructure of the films of this work are compromised by the reduced thermal budget employed, as expected. Anyway, the permittivities shown here are promising with regard the introduction of ferroelectric oxides as high-permittivity dielectrics for capacitance applications in future high-density memory generations (note that ε_r_ values of current SiO_2_ and Ta_2_O_5_ candidates are ~7 and ~22, respectively)^53^. The leakage current density of the PZT films shown in Fig. 5b is relatively low, with acceptable values at low voltages for memory operation. Higher values with respect to low-permittivity materials (i.e., the former single oxides) were measured in the films of this work at high voltages, although no resistance degradation was observed. The dielectric properties of these PZT films are indicative of a diminished crystallinity in the material due to a low thermal budget that detrimentally affects the size and connectivity of the ferroelectric grains. This means that films with an improved electrical response may be obtained at this temperature at the expense of e.g. longer annealing times (i.e., increasing the thermal budget), as inferred from previous Fig. 2c. To surpass the thermal limitations on the achievable crystallinity of low-temperature processed metal oxide thin films is the subject of current research in our group.

The ferroelectric hysteresis curve measured at room temperature in this film is shown in Fig. 5c. The compensated loop showing the real contribution to ferroelectric domain switching (up left), and the charge versus voltage response coming from capacity and conductivity, i.e. non-switching contribution, of the sample (down right) are also included. A remanent polarization (P_r_) of 10.0 μC cm^−2^ is obtained from the compensated loop of the 400 °C-annealed film, with a coercive field (E_c_) of 195 kV cm^−1^ (1.3 V for a 70 nm film thickness). The switching charge density of this film is within the values of FeRAM technology requirements predicted in the last ITRS report2 (8.5 μC cm^−2^), where the low-voltage operation is considered a key issue as well. State-of-art of latest FeRAM prototypes^54^ developed in the market demands power supply values of 1.5 V with 130 nm CMOS process technology. The ferroelectric properties of this sample, together with the reduced thermal budget employed, offer big potential gains for ferroelectric thin films in the demanding field of integration with silicon microelectronics. Low-temperature ferroelectrics (≤450 °C) may surpass the technology limits predicted for these materials with cell area decrease, thus succeeding the critical 130 nm node of most FeRAM commercial applications. The low preparation temperature of these PZT films is expected to significantly reduce the interdiffusion of elements between the ferroelectric layer and the semiconductor substrate, opening the door to a direct deposition on silicon (i.e., in the absence of insulating buffer layers). Thus, traditional logic devices based on ferroelectric materials would acquire renewed interest in terms of implementation and novel concept design. As an illustration, the detrimental effects typically associated with inorganic FeFETs (charge trapping and thermal instability at ferroelectric-semiconductor interface) could be overcome by this low-temperature PZT layer, lowering significantly the producction costs while mantaining the superior performance with respect to organic ferroelectric counterparts^55^,^56^.

**Figure 6:**
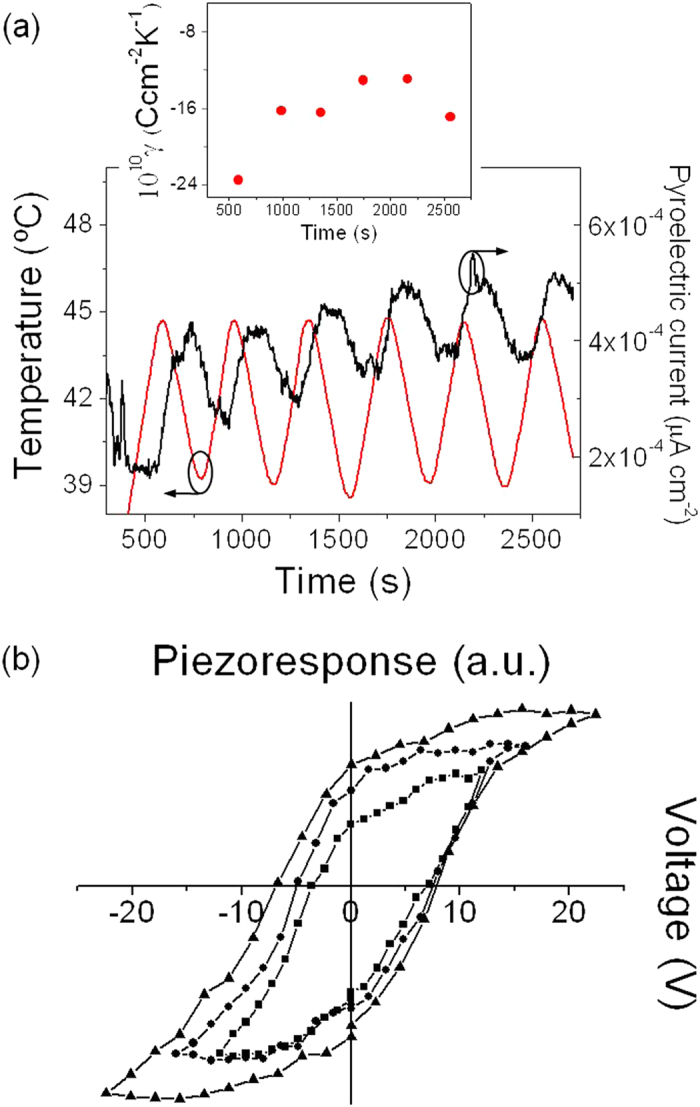
Multifunctional nature of the PZT thin film prepared from the *Seeded Photosensitive* solution at 400 °C. (**a**) Variation on the current density produced by an oscillating thermal wave after poling (inset shows the evolution of the pyroelectric coefficient with time). (**b**) Local piezoelectric response by successive hysteresis loops at different voltages.

The low-temperature films of this work also demonstrate pyroelectric and piezoelectric performance, as shown in Fig. 6, which broadens the application range of these active layers in electronic devices. Figure 6a depicts the current variations induced in this sample (400 °C annealed film) by the application of an oscillating thermal wave, yielding the evolution of the pyroelectric coefficient with time (see inset). The pyroelectric response of the film (~1.9 × 10^−9^ Ccm^−2^K^−1^), together with its long-term stability, supports its reliability for diverse applications in the field of thermal radiation detection^57^, such as gas sensors, thermal imaging or security detectors. For an optimal application, it is worth mentioning that PZT films of Ti-rich compositions would give better figures of merit due to their lower permittivities and dielectric losses in comparison to MPB PZT. For two-dimensional pyroelectric arrays fabricated on silicon, transistors are integrated into the cell to enable the optimum signal processing (switching, amplification) of the device. The reduced annealing temperature employed here, with a value below the maximum limit of standard metal interconnects, would facilitate the CMOS processing for the readout electronics carried out prior to the ferroelectric thin film processing and micromachining steps^58^. Demonstration of piezoelectric response in this film is additionally revealed by the local piezoelectric hysteresis loops of Fig. 6b. As miniaturization of microelectronic devices proceeds further, integration of high-performance piezoelectric layers with semiconductor circuits becomes increasingly important for compactness and performance reasons. Therefore, to control the thickness and hence resistivity of the metal silicides (Ni, Co, Ti) associated with the transistor, lower thermal budgets are demanded for advanced technologies beyond the 65 nm node^59^. The existence of piezoelectricity in MPB PZT thin films processed at a temperature of only 400 °C raises new opportunities for micro- and nanoelectromechanical systems (MEMS, NEMS)^60^, with increased sensitivity and optimum scalability. Particularly, ferroelectric films prepared by this low-temperature solution method may provide electronic systems integrators for MEMS/NEMS devices with higher performance, lower cost, and multiple sensor functions.

## Conclusions

This work demonstrates that ferroelectric thin films of Pb(Zr_0.52_Ti_0.48_)O_3_ (PZT) can be integrated into silicon computer chips at annealing temperatures below 450 °C, where the safe region for the CMOS process of microelectronics is currently settled. A low-cost and high-throughput fabrication technique based on a low-temperature solution method (photochemical solution deposition of seeded diphasic precursors) enables the growth of ferroelectric PZT layers on semiconductor substrates from 350 °C, the lowest temperature reported so far for this commercial ferro-piezoelectric composition at the morphotropic phase boundary. The morphology of the films is clearly influenced by the presence of the nanoseeds, which act as nucleation sites leading to uniform nanostructures constituted by fine grains (~30 nm) optimum for the fabrication of discrete capacitors with a low aspect ratio. Also, heterogeneous nucleation over the nanoseeds surface accounts for the contribution of randomly-oriented crystals in the perovskite films, in addition to the <111> preferred orientation promoted by nucleation at the substrate interface. Despite the crystal quality and microstructure of the films being compromised by the low thermal budget employed, the ferroelectric properties of the films of this work are competitive to the main requirements demanded for a practical application such as a memory device (FeRAM). The adequate values of remanent polarization (10.0 μC cm^−2^), relative permittivity (~90), and loss tangent (<10%) obtained, together with the demonstration of pyroelectric and piezoelectric performance, cover the ever increasing necessity for low-temperature inorganic ferroelectrics envisaged by the microelectronic industry in view of potential applications in micro- and nanoscale devices.

## Methods

### Synthesis of precursor solutions

The preparation of a precursor solution with nominal composition of Pb(Zr_0.52_Ti_0.48_)O_3_ (PZT) was carried out by a modified sol-gel method^61^. Lead acetate trihydrate, Pb(OOCCH_3_)_2_·3 H_2_O (Sigma-Aldrich, *ACS reagent*, ≥99%), was initially dissolved at room temperature in a mixture of solvents consisting of 1,2-propanediol, HOCH_2_CHOHCH_3_ (Sigma-Aldrich, *ACS reagent*, ≥99.5%), and acetic acid, CH_3_COOH (Sigma-Aldrich, 99.8%). Molar ratios with the Pb(II) ion of 5:1 and 0.1:1 were respectively used. To enhance the photosensitivity of the solution, zirconium n-propoxide, Zr(OC_3_H_7_)_4_ (Sigma-Aldrich, 70 wt% in 1-propanol), and titanium isopropoxide, Ti(OC_3_H_7_)_4_ (Sigma-Aldrich, 97%), were modified with acetylacetone, CH_3_COCH_2_COCH_3_ (Sigma-Aldrich, *reagent plus*, ≥99%), in a molar ratio of 2:1 to the respective Ti(IV) and Zr(IV) metal ions. The resulting mixture was added to the former Pb(II) solution, which after stirring led to a stable PZT precursor. No lead excess was employed through the whole synthetic procedure. The stock solution was diluted with ethanol, CH_3_CH_2_OH (Merck, *secco-Solv*, max. 0.01% H_2_O), to a final concentration of 0.2 M (*Ph solution*).

PZT seed nanopowders were obtained by a sol-gel process described elsewhere^62^. A stable suspension of these nanopowders in ethanol was achieved by the use of surfactants such as Dispex A40 (ammonium salt of polycarboxylic acid, Allied Colloids Ltd.) and/or Hypermer KD2 (polymeric surfactant, ICI Colour & Fine Chemicals). Perovskite nanoseeds were introduced from aliquots of this suspension in the *Ph solution* leading to a seeded diphasic photosensitive precursor with a 2 wt% concentration of seeds to the equivalent PZT solution (*PhS solution*). A high-intensity ultrasonic liquid processor (Sonics & Materials Inc, Vibra-CellTM Ultrasonic Processor) was used to disperse the seeds.

### Preparation of thin films

Films from the former solutions were deposited on Pt/Ti/SiO_2_/(100)Si substrates by dip-coating (KSV Intruments Ltd., KSV dip coater) using a withdrawal speed of 3 mm s^−1^, and then dried on a hot-plate at 150 °C for 600 s. Deposition and drying were repeated twice in order to growth films in the sub-100 nm regime (70 nm thickness). The gel layers were irradiated with UV light at 250 °C for 1 h in oxygen in a laboratory-scale prototype combining a high-intensity UV excimer lamp (Heraeus, BlueLight Excimer System, 222 nm wavelength) with a ultra-fast heating system (Watlow, Ultramic^TM^ 600 advanced ceramic heater). The photoactivated films were subsequently crystallized at different temperatures by rapid thermal processing (Jipelec, JetStar 100 T Processor) in oxygen using soaking times of 1 and 5 h. The temperature during irradiation and crystallization steps was controlled by the internal thermocouples of the respective equipments. External thermocouples on the surface of the films were also used to monitor the working temperature. Additionally, coatings on test samples (e.g., bare substrates) from liquid temperature lacquers (Omega Engineering Inc., Omegalaq) were applied to guarantee that the stated temperature of the crystallization step is not surpassed. The studies showed a temperature variation less than ±5 °C. A planar array of discrete capacitors was formed in the films by sputtering Pt top electrodes on their surface through a shadow mask.

### Characterization of materials

Photosensitivity of the precursor solutions was measured with a Varian Cary 50 Bio UV-visible spectrophotometer. Stability of the PZT nanoseeds with time in the *PhS solution* was studied by transmittance mode using a green light of 545 nm wavelength. The particle size distribution of the seeds dispersion was obtained by dynamic light scattering (DLS), using a Coulter LS 230 analyser. Temperature of the analysis was 25 ± 0.1 °C.

X-ray diffraction (XRD) under Bragg-Brentano (θ/2θ) configuration was used to analyze the crystallinity of the films. Measurements were performed in a Bruker D8 T2T SOLX diffractometer (Bruker AXS GmbH, Karlsruhe, Germany) with a Cu anode (λ_Cu_ = 1.54 Å). Surface morphology of the films was observed by scanning electron microscopy (SEM) in a Nova Nanosem 230 FEI Company instrument (FEI Company, Hillsboro, OR). X-ray photoelectron spectroscopy (XPS) was carried out in a Physical Electronics PHI 5700 spectrometer with nonmonochromatic Mg Kα radiation (1253.6 eV) and 29.35 eV constant pass energy. Analysis of the core level signals of O 1*s*, C 1*s*, Pb 4*f*, Zr 3*d*, Ti 2*p*, Pt 4*f*, and Si 2*p* with a multichannel detector was performed in the sample, using a 4 keV Ar^+^ bombardment at a current density of 1.8 μA cm^−2^. The in-depth scale of 3.1 nm min^−1^ was assumed to be equivalent to the sputter rate of SiO_2_ under the same sputter conditions. The film thickness was estimated from cross sectional SEM images and calculated by XPS using a 100 nm thick SiO_2_ layer as a reference.

Evaluation of the dielectric properties of the films was carried out with a HP 4194 A precision LCR meter using the built-in procedure (0.01 V oscillating voltage at 10 kHz) and with a Radiant Precision Premier II materials analyzer. Ferroelectric hysteresis loops were measured by means of virtual ground set up circuit, using a HP 8116 A pulse generator to produce sinusoidal electrical excitation with 1 kHz frequency. The compensated hysteresis loops were calculated as indicated in reference^63^. Pyroelectricity of the films was analyzed after poling the sample with a train of pulses. To calibrate the optimal pulse width and amplitude, switching current measurements were performed on the samples to assure more than 90% of switching. In the case of the 400 °C-annealed films, a train of pulses (1 s) of 25 μs width, 25 μs of separation and 7 V amplitude was used. Pyroelectric coefficients were calculated from the pyroelectric currents measured in a Keithley 6512 electrometer. These currents were obtained by the application of a triangular thermal wave of 5 × 10^−2^ Hz with an amplitude of 2 °C, using heating and cooling rates of ±1.8 °C. Piezoresponse force microscopy (PFM) measurements were performed in a commercial atomic force microscope (Multimode, Veeco) using Pt/Ir coated tips (EFM-20, Nanoworld, l = 225 μm, resonant frequency of ∼75 kHz, spring constant of ~2.8 N m^−1^).

## Additional Information

**How to cite this article**: Bretos, I. *et al*. Active layers of high-performance lead zirconate titanate at temperatures compatible with silicon nano- and microelecronic devices. *Sci. Rep*. **6**, 20143; doi: 10.1038/srep20143 (2016).
