# Corrigendum: Active layers of high-performance lead zirconate titanate at temperatures compatible with silicon nano- and microelectronic devices

**DOI:** 10.1038/srep22499

**Published:** 2016-03-07

**Authors:** Iñigo Bretos, Ricardo Jiménez, Monika Tomczyk, Enrique Rodríguez-Castellón, Paula M. Vilarinho, M. Lourdes Calzada

Scientific Reports 6: Article number: 2014310.1038/srep20143; published online: 02032016; updated: 03072016

The original version of this Article contained an error in the title of the paper, where the word “microelectronic” was incorrectly given as “microelecronic”. This has now been corrected in the PDF and HTML versions of the Article.

